# Dual role of Ca^2+^-activated Cl^−^ channel transmembrane member 16A in lipopolysaccharide-induced intestinal epithelial barrier dysfunction in vitro

**DOI:** 10.1038/s41419-020-2614-x

**Published:** 2020-05-29

**Authors:** Jingru Sui, Chi Zhang, Xuesheng Fang, Jianwen Wang, Yu Li, Jingyu Wang, Liang Wang, Jianyi Dong, Zijuan Zhou, Changyi Li, Jun Chen, Tonghui Ma, Dapeng Chen

**Affiliations:** 1 Comparative Medicine, Dalian Medical University, 116044 Dalian City, Liaoning Province China; 2 College of Basic Medical Science, Dalian Medical University, 116044 Dalian City, Liaoning Province China; 3 Holistic Integrative Medicine, Nanjing University of Chinese Medicine, Nanjing City, Jiangsu Province China

**Keywords:** Target identification, Physiology

## Abstract

Dysfunction of intestinal epithelial Cl^−^ currents and channels have previously been reported in inflammatory intestinal diseases. However, the expression and function of the newly identified Ca^2+^-activated Cl^−^ channel transmembrane member 16A (TMEM16A) in the intestinal epithelium is unclear. In this study, we investigated the effects of TMEM16A on intestinal epithelial barrier function in vitro. Intestinal epithelial barrier dysfunction was modeled by lipopolysaccharide (LPS)-induced cell damage in intestinal epithelial IEC-6 cells and the effects of TMEM16A knockdown and overexpression on cell apoptosis and tight junctions were studied. Corresponding mRNA and protein expression levels were measured by quantitative real-time polymerase chain reaction, western blotting, and immunofluorescence analysis, respectively. TMEM16A expression was significantly increased by LPS, possibly via a process involving the transcription factor nuclear factor-κB and both Th1 and Th2 cytokines. Low- and high-dose LPS dysregulated tight junctions (high-myosin light-chain kinase expression) and cell apoptosis-dependent cell barrier dysfunction, respectively. TMEM16A aggravated cell barrier dysfunction in IEC-6 cells pretreated with low-dose LPS by activating ERK1/MLCK signaling pathways, but protected against cell barrier dysfunction by activating ERK/Bcl-2/Bax signaling pathways in IEC-6 cells pretreated with high-dose LPS. We concluded that TMEM16A played a dual role in LPS-induced epithelial dysfunction in vitro. The present results indicated the complex regulatory mechanisms and targeting of TMEM16A may provide potential treatment strategies for intestinal epithelial barrier damage, as well as forming the basis for future studies of the expression and function of TMEM16A in normal and inflammatory intestinal diseases in vivo.

## Introduction

Specialized epithelial cells form a biochemical and physical barrier that separates mammals from the external environment. The gastrointestinal tract is the largest such barrier, with direct connections with commensal bacteria and impacts on the development and function of the mucosal immune system^1,2^. Microbial colonization following disruption of epithelial or immune cell homeostasis increases the risk of infection and inflammation^3,4^. Epithelial barrier dysfunction results in translocation of the bacteria, thus, increasing the risk of inflammation and inflammatory bowel disease (IBD)^5,6^. Increasing evidence has also indicates that loss of intestinal barrier function contributes to many other diseases, including chronic viral infections, diabetes, rheumatoid arthritis, and multiple sclerosis^7–10^.

The intestinal epithelial barrier is maintained by many factors, including secreted and transported intestinal epithelial cell defenses (mucins (MUCs), antimicrobial proteins, and IgA)^11,12^, apoptosis/proliferation of epithelial cells^13^, and cell junctions, including adherens and tight junctions^14^. Intestinal barrier function is mainly defined by the permeability of the tight junctions in the intact epithelium^15^. Intestinal epithelial tight junctions are areas where the membranes of two adjacent cells join to form a barrier that prevents molecules from passing through and stops membrane proteins from moving around^16,17^. However, epithelial cell apoptosis results in loss of barrier function, regardless of the presence of tight junctions, and is referred to as apoptosis-related barrier dysfunction. The differentiation of intestinal mucosal epithelial cells is a dynamic process that depends on the balance between epithelial cell apoptosis and proliferation^18,19^. Apoptosis plays an important role in the expulsion of damaged cells, while excessive apoptosis occurs under pathological conditions, such as IBD^20^. Ca^2+^-activated Cl− channel transmembrane member 16A (TMEM16A, also known as anoctamin-1 or dog1) was newly identified as a candidate Ca^2+^-activated Cl− channel in 2008^21^. TMEM16A is expressed in intestinal epithelial cells and controls the apical outflux of Cl−, which in turn aids fluid transport^22,23^. TMEM16A has been shown to be involved in many diseases, including cancer, hypertension, and cystic fibrosis^24–26^, and TMEM16A activation is also involved in rotavirus toxin NSP4-induced diarrhea^27^. However, the expression and function of TMEM16A in the intestinal epithelium is currently controversial. Some researchers showed that TMEM16A was necessary for ATP-dependent mucus secretion in the intestine^28,29^, while others found no involvement of TMEM16A in electrogenic calcium-activated anion transport and mucus homeostasis^30^. TMEM16A alleviates lipopolysaccharide (LPS)-induced inflammatory responses in human lung epithelial cells and involved in alveolar fluid clearance^31,32^, while inhibiting TMEM16A is of paramount importance to induce apoptosis in human prostate carcinoma^33^.

We therefore aimed to clarify the expression and functional role of TMEM16A in intestinal epithelial cells. In this study, we examined the effects of TMEM16A on cell apoptosis and tight junction barrier function in intestinal epithelial cells in vitro, to avoid potential interference from intestinal bacterial, intestinal mucus, and other factors. We used the rat intestinal epithelial IEC-6 cell line and established a cell barrier dysfunction model by LPS^34^.

## Materials and methods

### Reagents

TMEM16A antibodies (ab53213), MLCK antibodies (ab76092), cleaved caspase3 antibodies (ab2302), Bcl-2 antibodies (ab59348), and Bax antibodies (ab53154) were bought from Abcam (Hong Kong) Ltd. (Hong Kong, China). The TMEMD16A antibodies (14476S), phosphorylated ERK1/2 antibodies (#4370) and ERK1/2 antibodies (#4695), were bought from Cell Signaling (Boston, USA). The TMEMD16A antibodies (12652-I-AP) were bought from Proteintech Group (Chicago, USA). The rat intestinal epithelial cell line IEC-6 cells were bought from cell bank of Shanghai Institute (Shanghai, China). BrdU kit (ab126556) was obtained from Abcam (Hong Kong) Ltd. (Hong Kong, China). The cells used in this study were evaluated before experiment. Unless otherwise indicated, chemicals were obtained from Sigma-Aldrich (St. Louis, MO, USA).

### Animals

Sixty C57BL/6 mice (male, weighing 18–20 g) were obtained from the experimental animal center at Dalian Medical University (Certificate of Conformity: No. SYXK (Liao) 2013-0006). The experimental protocol was approved by the Animal Care and Ethics Committee of Dalian Medical University, Liaoning, China, and carried out in accordance with the “Principles of Laboratory Animal Care and Use in Research” guidelines (State Council of China, 1988). The animal protocol was designed to reduce pain and discomfort to the animals. The mice were acclimatized to laboratory conditions (23 °C, 12 h/12 h light/dark, 50% humidity, ad libitum access to food and water) for two weeks prior to experimentation. The mice were housed at a density of one per cage. Animal studies are reported in compliance with the ARRIVE guidelines^35^.

Two colitis models including dextran sodium sulfate (DSS)-induced and 2,4,6-trinitrobenzene sulfonic acid (TNBS)-induced colitis models were used in this study to investigate the expression of TMEM16A in intestinal epithelium in inflammatory intestine diseases. The mice-DSS colitis model was established by free drinking water containing 2.5% DSS for seven days^36^. The mice-TNBS colitis model was induced as described previously^37^. Briefly, rats were fasted for 24 h with free access to drinking water. A catheter was inserted through the anus about 8 cm proximal to the anal verge under urethane anesthesia. The colon was then infused with 1 mL of TNBS dissolved in ethanol (50% v/v) at a dose of 125 mg/kg. Distal colonic epithelium was harvested for biochemical studies.

### Cell culture

Rat intestinal IEC-6 epithelial cells were maintained at 37 °C in a 5% CO_2_ environment. The culture medium consisted of dulbecco’s modified eagle’s medium (DMEM) with 4.5 mg/mL glucose, 50 U/mL penicillin, 50 U/mL streptomycin, 4 mM glutamine, 25 mM HEPES, and 10% fetal bovine serum. Cells were cultured in standard atmosphere until confluence for following experiments. Both fetal bovine serum and DMEM were purchased from Invitrogen (Waltham, MA, USA). The transepithelial electrical resistance (TER) of cultured IEC-6 cells was examined using an epithelial voltohmmeter^38^. A barrier dysfunction cellular model was established in IEC-6 cells exposed to LPS. IEC-6 cells were transfected using Lipofectamine 3000 (Invitrogen) with TMEM16A targeted (sense sequence, CCG GAG CAC AAU AGU UCU AUT T), and NF-κB p65 targeted (sense sequence, GUG GGC CUU AAU AGC CAU ATT) or control small-interfering RNA (siRNA) oligos (Dharmacon, Lafayette, CO, USA). Confluence monolayers were treated with LPS (1 μg/mL) 36 h later. The efficiency of gene silencing was confirmed by western blotting or immunofluorescence. The cells were transfected with adenovirus containing TMEM16A and green fluorescent protein (GFP) (AdCMV/TMEM16A-GFP) (GenePharma, Suzhou, China) to stimulate the expression of TMEM16A. The cell proliferation was determined by CCK-8 analysis according to the manufacture’s suggestions. The CCK-8 kit was bought from Wanleibio (Shenyang, China).

### Flow cytometry analysis

The apoptosis of IEC-6 cells was measured by flow cytometry. IEC-6 cells were disposed by 12 h incubation with 10 μg/mL LPS in the condition of knocking down and overexpression of TMEM16A, respectively. Untreated IEC-6 cells were served as a normal control. The cells were collected after 5 min digestion using 0.5 mL 0.25% cold trypsin without EDTA, washed with washing buffer for three times. Counted and balanced the number of cells each group, and then stained with fluorescein isothiocyanate (FITC)-Annexin V and propidium iodide (PI) according to the manufacturer’s protocol (Annexin V-FITC Apoptosis Detection Kit, BD Pharmingen, USA). Apoptotic cells (Annexin V-positive and PI-negative) were then quantified by flow cytometry using the BD analysis program (BD Pharmingen, USA).

### TMEM16A expression analysis

Total RNA isolation and real-time PCR for RNA quantification were performed, and the housekeeping gene GAPDH served as an internal control. The messenger RNA (mRNA) levels of TMEM16A were normalized to the mRNA levels of the housekeeping gene GAPDH. Proteins were extracted from cells and separated by SDS-PAGE (10%) and transferred to polyvinylidene difluoride membranes. Membranes were blotted for specific antibodies. The blots were developed using an enhanced chemiluminescence method (GE Healthcare). Quantification was performed by densitometric analysis of specific bands on the immunoblots using a Multi Spectral imaging system (UVP, Cambridge, UK).

### Immunofluorescence and assessment of inflammation

IEC-6 cells were cultured in standard condition for 12 h before the experiment. Cells were fixed with 4% paraformaldehyde (SenBeiJia, Nanjing, China) for 20 min. Then cells were incubated in 0.2% Triton X-100 (Solarbio) for 5 min. Next, the nonspecific protein binding sites were blocked with 3% bovine serum albumin (BSA)-phosphate-buffered saline ((PBS); ZSGB-BIO, Beijing, China) at 37 °C for 1 h. Then cells were incubated overnight with related primary antibody at 4 °C. After washing with PBS for 15 min, cells were incubated for 1 h at 37 °C with an anti-rabbit secondary antibody (SA00003-2, Proteintech). Cells were then treated with prolong^TM^ gold antifade reagent with DAPI (Invitrogen) and were stored from night. Images were verified using the confocal microscope (FV1000PME, Japan). Colon sections were processed in the same way as above, and the images were verified by fluorescence microscope. Furthermore, the inflammation caused by DSS or TNBS was assessed by hematoxylin and eosin (HE).

### Statistical analysis

The animal experiments, in vitro experiments, and data analyses were conducted according to a single-blind study design. Data were compared among three or more groups using a one-way ANOVA, and between two groups using Student’s *t*-tests. Data were expressed as the mean ± SD. Data were normally distributed and each group showed similar variances. Further evaluations were carried out using Kruskal–Wallis rank sum tests. All experiments were repeated at least three times and a *p-*value < 0.05 was considered statistically significant.

## Results

### LPS stimulated TMEM16A expression

**Fig. 1:**
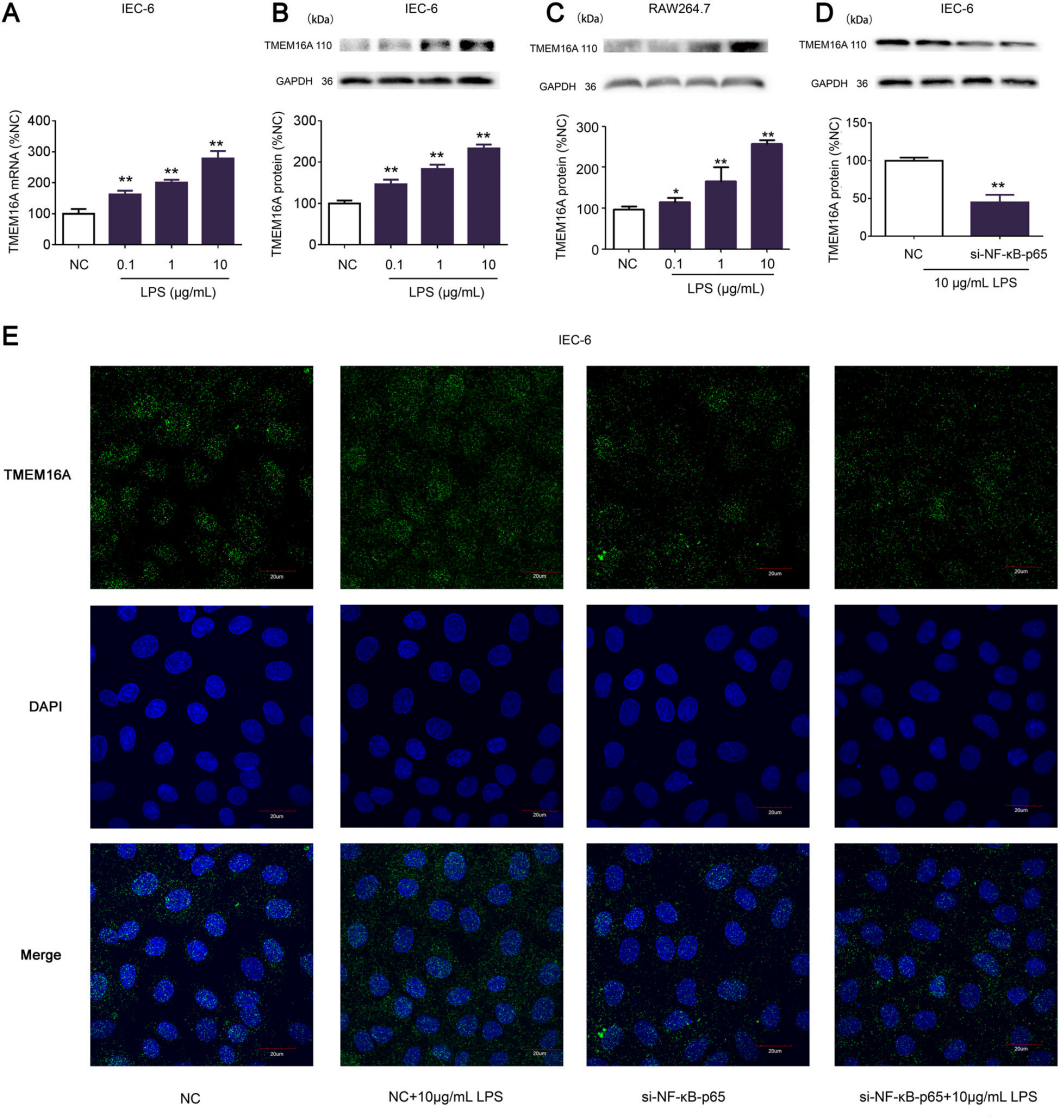
LPS increased TMEM16A expression. **a**, **b** Expression analysis of TMEM16A by qRT-PCR and Western blotting in IEC-6 cells incubated with LPS. (*n* = 3/group). **c** Expression analysis of TMEM16A by Western blotting in RAW264.7 cells incubated with LPS. (*n* = 3/group) **d**, **e** Effects of siRNA-targeting NF-κB p65 on TMEM16A expression in IEC-6 cells incubated with LPS studied by Western blotting and confocal microscope (green fluorescence), scale bar = 20 μm. (*n* = 6/group). Data are expressed as the mean ± SD. Values in the normal control (NC) group are set to 100% and other values are given relative to those in the NC group, ^*^p < 0.05, ^**^p < 0.01 compared with NC group. si-NF-κB p65, in the presence of siRNA-targeting NF-κB p65.

mRNA and protein expression levels of TMEM16A in IEC-6 cells were significantly increased by 0.1, 1, and 10 μg/mL LPS (Fig. 1a, b). We compared these results in IEC-6 cells with the macrophage cell line RAW264.7 and showed that TMEM16A protein expression was also significantly increased by 0.1, 1, and 10 μg/mL LPS in RAW264.7 cells (Fig. 1c). As we were specifically interested in the expression and function of TMEM16A in intestinal epithelial cells, we chose IEC-6 cells for subsequent studies.

NF-κB p65 plays an important role as a transcription factor during the development of inflammation. We therefore determined if NF-κB p65 was involved in the LPS-induced expression of TMEM16A. The LPS (10 μg/mL)-induced increase in TMEM16A expression was significantly inhibited by siRNA-targeting NF-κB p65 (Fig. 1d). In the presence of 10 μg/mL LPS, cell number was decreased; we select the area with similar cell number to study with the help of the confocal microscope. Immunofluorescence results (Fig. 1e) also showed that 10 μg/mL LPS significantly stimulated the expression of TMEM16A, and this stimulation was significantly inhibited by siRNA-targeting NF-κB p65. NF-κB p65 has been identified as a candidate transcription factor in three databases (Supplementary Tables). These results indicated that LPS could increase TMEM16A expression, and that NF-κB p65 may be a transcription factor affecting TMEM16A expression.

### Effects of TMEM16A on TER in IEC-6 cells

**Fig. 2:**
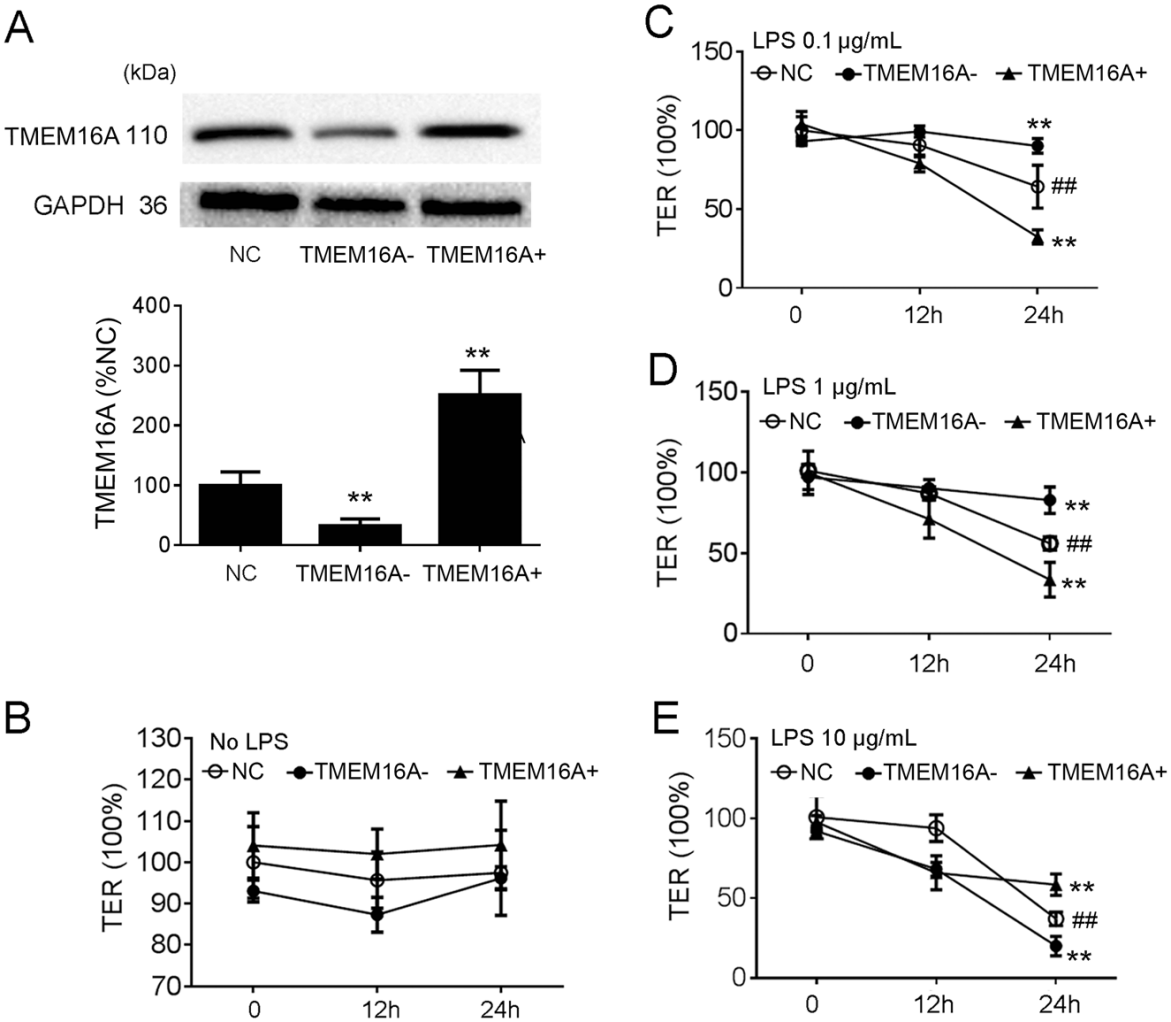
Effects of TMEM16A on IEC-6 cell transepithelial electrical resistance (TER). Cells were cultured for the first seven days to form a monolayer barrier. Media were changed every three days before the experiment. On corresponding time points, media contained siRNA-targeting TMEM16A (TMEM16A–) or adenovirus stimulating TMEM16A (TMEM16A+) in the absence and presence of LPS was added. On day 8, the TER in corresponding groups was measured. **a** The effects of TMEM16A- and TMEM16A + was confirmed prior to the experiments. (*n* = 6/group). **b**, **c**, **d**, **e** Effects of TMEM16A on IEC-6 cell TER was studied in the presence of no LPS), 0.1 μg/mL LPS, 1 μg/mL LPS, 10 μg/mL LPS, respectively. (*n* = 6/group). Values in the normal control (NC) group are set to 100% and other values are given relative to those in the NC group. ^##^*p* < 0.01 compared with that in NC group, ^**^p < 0.01 compared with that in NC group at 24 h.

The current results confirmed that TMEM16A expression was significantly decreased by transfection with its corresponding siRNA, and was significantly increased by adenovirus containing TMEM16A (AdCMV/TMEM16A-GFP) (Fig. 2a). We therefore examined the role of TMEM16A in LPS-induced intestinal barrier dysfunction in IEC-6 cells with knockdown or overexpression of TMEM16A. The TER of normal IEC-6 cells was not significantly affected by TMEM16A knockdown or overexpression (Fig. 2b). However, the TER was significantly decreased in cells incubated with 0.1, 1, and 10 μg/mL LPS for 24 h, indicating epithelial barrier dysfunction (Fig. 2c–e). The decreased TER was enhanced by siRNA-targeting TMEM16A, but aggravated by overexpression of TMEM16A in IEC-6 cells incubated with relatively low-dose LPS (0.1 and 1 μg/mL) (Fig. 2c). However, the IEC-6 cell barrier dysfunction induced by relatively high-dose LPS (10 μg/mL) was alleviated by overexpression of TMEM16A, but aggravated by siRNA-targeting TMEM16A (Fig. 2e). These results suggested that TMEM16A aggravated cell barrier dysfunction induced by low-dose LPS, but protected against cell barrier dysfunction caused by high-dose LPS. TMEM16A, thus, played different roles in low- and high-dose LPS-induced cell barrier dysfunction, respectively.

### Mechanisms underlying dual role of TMEM16A in LPS-induced cell barrier dysfunction

**Fig. 3:**
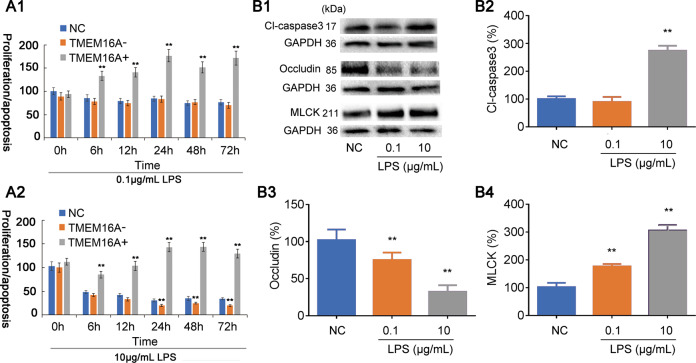
Mechanisms underlying the dual role of TMEM16A in LPS induced cell barrier dysfunction. **a** MTT and BrdU analysis was carried out to test the ration of cell proliferation and apoptosis (proliferation/apoptosis) at different time points. Effects of TMEM16A on IEC-6 cell proliferation/apoptosis incubated with relative low dose LPS (0.1 μg/mL LPS) (a1) and high dose LPS (10 μg/mL LPS) (a2). (*n* = 6/group). **b** Representative (b1) and statistical results of expression of cleave caspase3 (Cl-caspase3) (b2), occludin (b3), and MLCK (b4) in low and high dose LPS incubated IEC-6 cells. (*n* = 6/group). Data are expressed as the mean ± SD. Values in the normal control (NC) group are set to 100% and other values are given relative to those in the NC group, **p < 0.01 compared with NC group.

We studied the mechanisms underlying the dual regulatory effects of TMEM16A by examining cell proliferation, apoptosis, and tight junctions in epithelial barrier dysfunction induced by low- and high doses of LPS. MTT and BrdU assay kit were used to investigate cell apoptosis and proliferation, respectively, and the proliferation/apoptosis ration was calculated. Results showed that IEC-6 cells incubated with low-dose LPS (0.1 μg/mL) proliferated slightly from 0 to 24 h. IEC-6 cell proliferation/apoptosis from 24 to 72 h was significantly stimulated by overexpression of TMEM16A, but was unaffected by siRNA-targeting TMEM16A (Fig. 3a1). Cell proliferation/apoptosis was significantly decreased from 24 to 72 h in IEC-6 cells incubated with high-dose LPS (10 µg/mL). High-dose LPS-induced IEC-6 cell proliferation/apoptosis was significantly inhibited by overexpression of TMEM16A and aggravated by siRNA-targeting TMEM16A (Fig. 3a2). These results indicated that LPS-induced IEC-6 cell barrier dysfunction involved two mechanisms: one induced by low-dose LPS that were mainly mediated by tight junction dysfunction but not cell apoptosis, and another induced by high-dose LPS that were mainly mediated by cell apoptosis. TMEM16A thus exerted a dual role in the regulation of LPS-induced cell barrier dysfunction in line with these different mechanisms.

We further studied the mechanisms underlying the dual regulation of LPS-induced cell barrier dysfunction by TMEM16A by evaluating the changes in expression levels of the apoptosis marker cleaved caspase3, tight junction protein occludin, and MLCK (Fig. 3b1–b4). Cleaved caspase3 levels were not significantly influenced in IEC-6 cells incubated with low-dose LPS, while occludin was significantly decreased and MLCK was significantly increased, confirming that low-dose LPS-induced epithelial barrier dysfunction involved tight junction dysregulation. In contrast, cleaved caspase3 and MLCK were significantly increased and occludin was significantly decreased in IEC-6 cells incubated with high-dose LPS, indicating that high-dose LPS induced epithelial barrier dysfunction by both tight junction dysregulation and cell apoptosis. It is believed that cell apoptosis may play a more important role in epithelial barrier dysfunction than tight junction dysregulation.

### TMEM16A in low-dose LPS-induced tight junction dysfunction

**Fig. 4:**
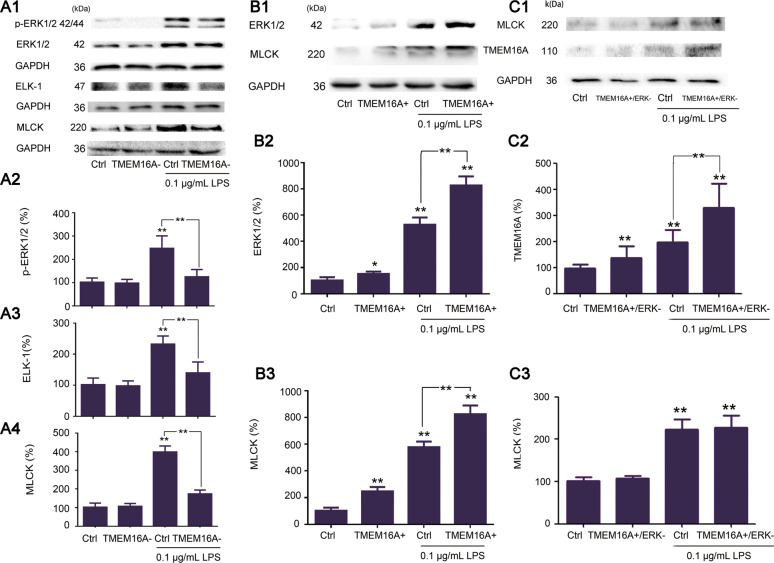
Effects and mechanisms of TMEM16A on LPS induced tight junction dysfunction. Cells were cultured for 24 h with media contained siRNA targeting TMEM16A (TMEM16A‑), ERK1(ERK1‑) or adenovirus stimulating TMEM16A (TMEM16A+) in the presence and absence of LPS. **a** Effects of TMEM16A‑ on low dose LPS (0.1 μg/mL) induced corresponding protein expressions in IEC-6 cells. Representative (a1) and statistical results of expression of p-ERK1/2 (a2), ELK-1 (a3), and MLCK (a4) were shown (*n* = 6/group). **b** Effects of TMEM16A+ on low dose LPS (0.1 μg/mL) induced corresponding protein expressions in IEC-6 cells. Representative (b1) and statistical results of expression of ERK1/2 (b2) and MLCK (b3) were shown (*n* = 6/group). **c** Effects of TMEM16A+ with siRNA targeting ERK1 (ERK-) on low dose LPS (0.1 μg/mL) induced corresponding protein expressions in IEC-6 cells. Representative (c1) and statistical results of expression of TMEM16A (C2) and MLCK (C3) were shown (*n* = 3/group). Data are expressed as the mean ± SD. Values in the normal control (NC) group are set to 100% and other values are given relative to those in the NC group, **p < 0.01 compared with corresponding group or as indicated.

MLCK expression was significantly increased in IEC-6 cells incubated with low-dose LPS compared with normal controls, indicating tight junction dysfunction. This LPS-induced increase in MLCK expression was significantly inhibited by siRNA-targeting TMEM16A (Fig. 4a1 and a4). Expression levels of p-ERK1/2, ERK1/2, and ELK-1 were also significantly increased in low-dose LPS-incubated IEC-6 cells, and these increases were significantly inhibited by siRNA-targeting TMEM16A (Fig. 4a1–a3). In addition, ERK1/2 and MLCK expression were further stimulated by overexpression of TMEM16A in the presence of low-dose LPS (Fig. 4b1–b3).

**Fig. 5:**
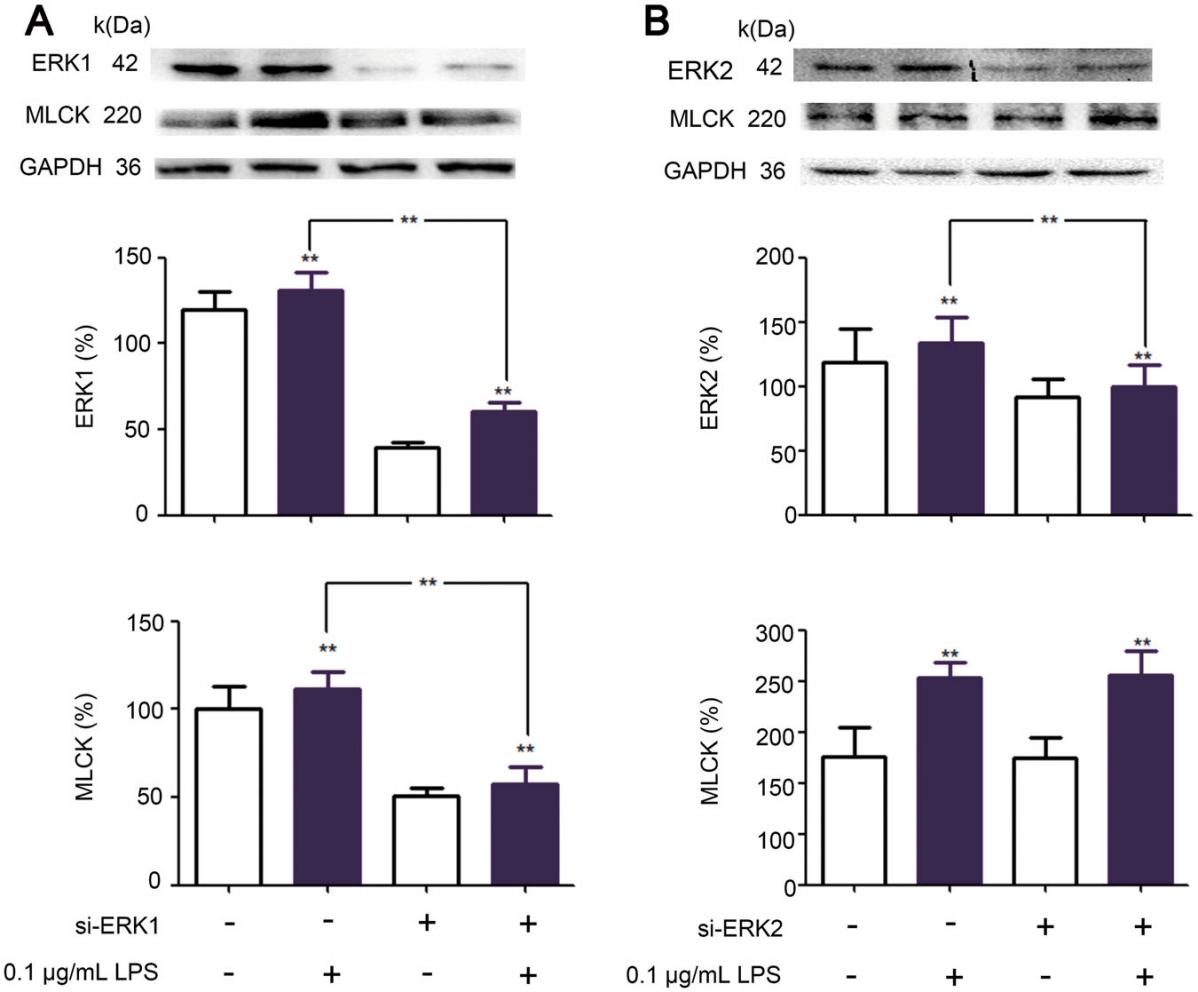
Effects of siRNA targetting ERK1(siERK1) and ERK2 (siERK2) on low dose (0.1 μg/mL) LPS induced tight junction barrier dysfunction. **a**, **b** The results with siERK1 and siERK2, respectively. Data are expressed as the mean ± SD. Values in the control group are set to 100% and other values are given relative to those in the control group. (*n* = 6/group). **p < 0.01 compared with corresponding control (same color) group or as indicated.

To confirm the involvement of ERK1/2 in the TMEM16A-induced increase of MLCK expression in the presence of low-dose LPS, we examined the effects of coincident knockdown of ERK1 and overexpression of TMEM16A. The increased expression of MLCK was not significantly enhanced by overexpression of TMEM16A together with siRNA-targeting ERK1 (Fig. 4c1–c3). siRNA-targeting ERK2 did not significantly affect the expression of MLCK in IEC-6 cells incubated with low-dose LPS (Fig. 5), and we, therefore, did not examine the effects of coincident knockdown of ERK2 and overexpression of TMEM16A. Overall, these results indicated that TMEM16A could stimulate MLCK expression by activating the TMEM16A/ERK1-ELK-1 signaling pathway, finally leading to tight junction barrier dysfunction in low-dose LPS-incubated IEC-6 cells.

### TMEM16A in high-dose LPS-induced cell apoptosis

**Fig. 6:**
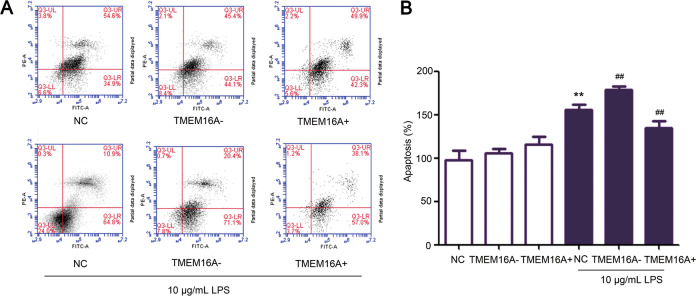
Effects of TMEM16A on LPS induced IEC-6 cell apoptosis measured by flow cytometry. Cells were cultured for 24 h with media contained siRNA targeting TMEM16A (TMEM16A‑) or adenovirus stimulating TMEM16A (TMEM16A+) in the presence and absence of high dose LPS (10 μg/mL). (*n* = 6/group). Representative (**a**) and statistical results (**b**) were shown. Data are expressed as the mean ± SD. Values in the normal control (NC) group are set to 100% and other values are given relative to those in the NC group, **p < 0.01 compared with NC group. ##*p* < 0.01 compared with corresponding TMEM16A‑ or TMEM16A+ group.

**Fig. 7:**
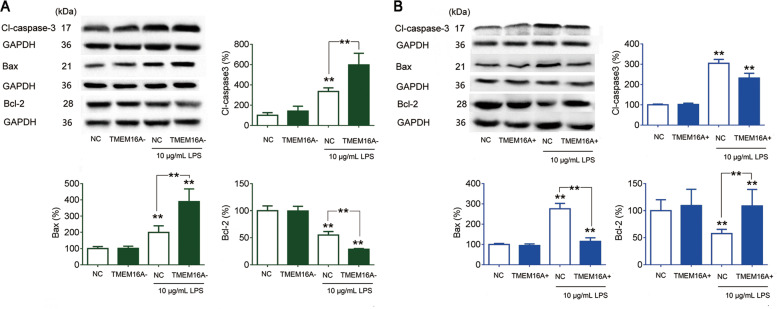
Effects and mechanisms of TMEM16A on LPS-induced IEC-6 cell apoptosis. Cells were cultured for 24 h with media contained siRNA-targeting TMEM16A (TMEM16A–) or adenovirus stimulating TMEM16A (TMEM16A+) in the presence and absence of high-dose LPS. **a** Effects of TMEM16A- and TMEM16A + on high-dose (10 μg/mL) LPS induced corresponding protein expressions in IEC-6 cells. (*n* = 3/group). **b** Effects of TMEM16A– and TMEM16A+ on high-dose (10 μg/mL) LPS induced corresponding protein expressions in IEC-6 cells. (*n* = 3/group). Data are expressed as the mean ± SD. Values in the normal (NC) group are set to 100% and other values are given relative to those in the NC group, ^**^p < 0.01 compared with corresponding control (same color) group or as indicated.

Cell apoptosis was significantly increased in IEC-6 cells incubated with high-dose LPS compared with normal controls, and this increase was significantly aggravated by siRNA-targeting TMEM16A and blocked by overexpression of TMEM16A, respectively, as demonstrated by preliminary flow cytometry analysis (Fig. 6). Cleaved caspase3 and Bax were significantly increased while Bcl-2 was significantly decreased in IEC-6 cells incubated with high-dose LPS, compared with controls (Fig. 7a). High-dose LPS induced increases in apoptosis markers were significantly aggravated by siRNA-targeting TMEM16A (Fig. 7a), and the high levels of cleaved-caspase3 and Bax were further reduced by overexpression of TMEM16A, while the low expression of Bcl-2 was further stimulated by overexpression of TMEM16A in the presence of high-dose LPS (Fig. 7b). Overall, these results indicated that TMEM16A protected against high-dose LPS-induced cell apoptosis by activating the ERK1/2 signaling pathway.

### TMEM16A expression in colon epithelium induced by DSS and TNBS

**Fig. 8:**
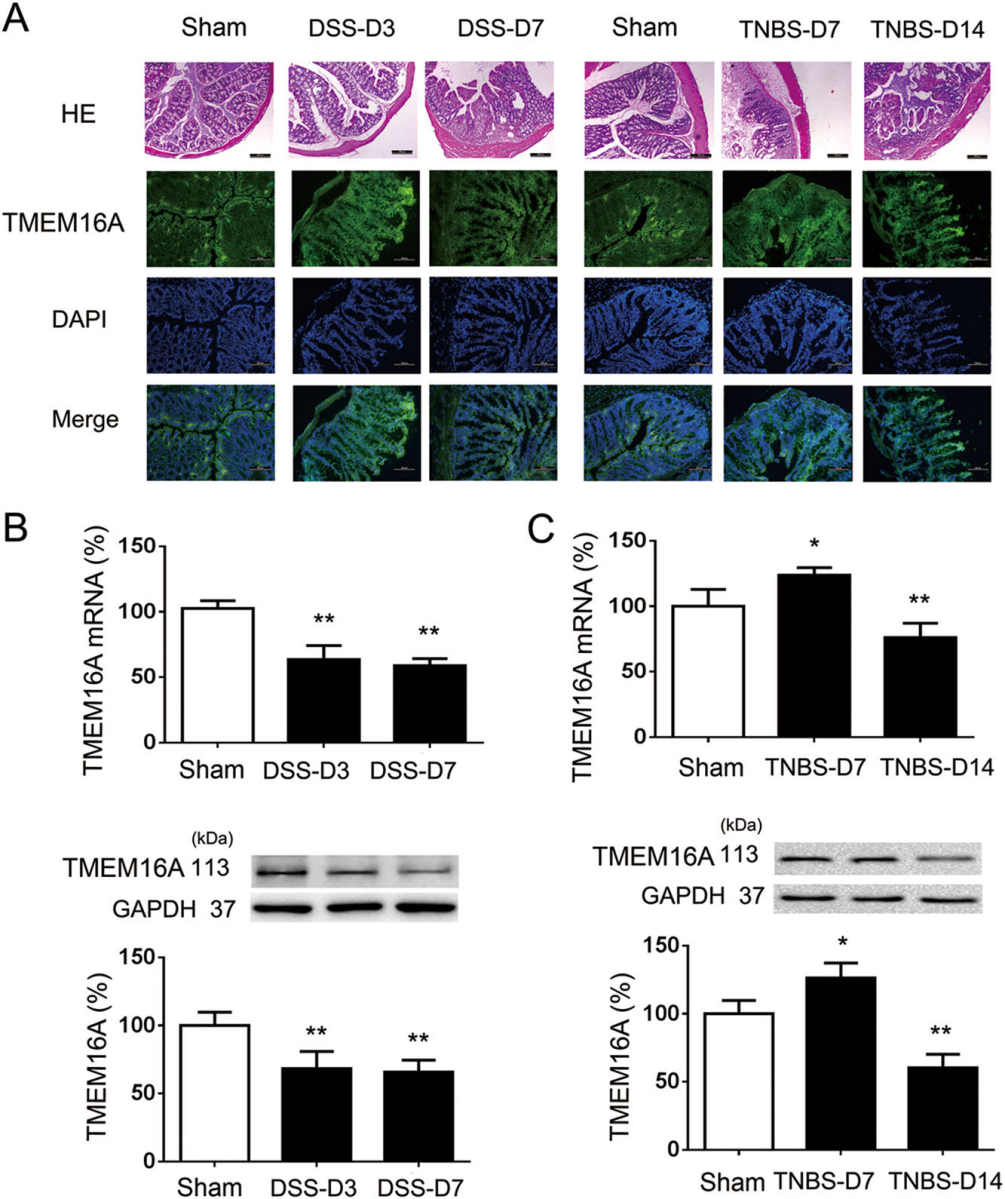
The expressions of TMEM16A in sham and colitis mice intestinal epithelium. **a** HE (×100) and expression of TMEM16A analyzed by immunofluorescence, scale bar = 200 μm. (*n* = 6/group). **b** mRNA and protein expressions of TMEM16A in DSS colitis. **c** mRNA and protein expressions of TMEM16A TNBS colitis. (*n* = 6/group). Data are expressed as the mean ± SD. Values in the sham group are set to 100% and other values are given relative to those in the sham group, ^*^p < 0.05, ^**^p < 0.01 compared with sham group.

In addition to examining the expression of TMEM16A in IEC-6 cells in vitro, we also investigated its expression in normal and pathologically inflamed intestine in vivo. We examined TMEM16A expression at different time points in DSS- and TNBS-colitis in mice, respectively. HE staining showed morphological changes in the colon after DSS and TNBS treatment. Furthermore, TMEM16A mRNA and protein levels were significantly decreased in DSS-colitis colonic epithelium on days 3 and 7, compared with normal controls (Fig. 8a, b), while levels in TNBS-colitis colonic epithelium were significantly increased on day 7 and significantly decreased on day 14 (Fig. 8a, c). We evaluated TMEM16A expression in the colonic epithelium using three kinds of antibodies, and demonstrated that TMEM16A protein was expressed in the colonic epithelium at a low level.

## Discussion

In this study, we examined the role of TMEM16A in LPS-induced IEC-6 cell barrier dysfunction. TMEM16A expression was significantly increased in IEC-6 cells exposed to LPS, and this increase was associated with both tight junction dysfunction and anti-apoptosis effects through activation of the MAPK/ERK1/2 signaling pathway. TMEM16A was involved in LPS-induced cell barrier dysfunction by activating both the ERK1/MLCK and ERK/BCL-2 signaling pathways.

TMEM16A expression was previously shown to be increased by bacterial components and Th2 cytokines in respiratory system diseases, and to aid the secretion of MUCs, especially MUC5AC and MUC5B^39–41^. In contrast to the respiratory system, the main MUC in the gut is MUC2^42^. The expression and function of TMEM16A in the normal and diseased intestinal epithelium remains controversial. Dysfunction of Cl^−^ secretion has been observed in intestinal inflammation, and TMEM16A has been identified as the primary mediator of Ca^2+^-activated Cl^−^ secretion in the colonic epithelium^43,44^. TMEM16A protein levels were decreased in a DSS-induced colitis model, and the authors predicted that the colitis was caused by downregulation of TMEM16A due to the stimulation of Th2 cytokines by DSS^45^. However, the mechanism responsible for mediating the downregulation of TMEM16A is unclear. Our results in Fig. 8 also confirmed that intestinal epithelial TMEM16A expression was decreased in DSS colitis model, while TMEM16A expression was increased in 7-day-TNBS colitis model. TMEM16A locates in basolateral side of the epithelium mainly, while TMEM16A also exists in apical membrane of goblet cells and enterocytes^46^. Therefore, the function of TMEM16A in inflammatory intestinal epithelium may be complicated due to its location and expression.

In contrast, some researchers found that TMEM16A was barely detectable in colon samples^30^. Our results also showed low expression of TMEM16A protein in colonic epithelium; however, it was easier to detect TMEM16A protein in the distal colon than in other gut regions such as the stomach, small intestine, and proximal colon (Supplementary Fig. 1B). However, TMEM16A was highly expressed in rat intestinal IEC-6 cells, mouse macrophage RAW264.7 cells, human intestine cancer Caco-2 cells, and mouse intestinal MODE-K cells in vitro (Supplementary Fig. 1A).

The function of TMEM16A in the intestine is still unclear, especially in terms of its effects on MUC secretion. We examined the effects of TMEM16A on cell barrier function in vitro and revealed that it played a dual role in LPS-induced epithelial barrier dysfunction: TMEM16A aggravated epithelial barrier damage induced by low-dose LPS through stimulating the ERK1/MLCK signaling pathway, and conversely protected against high-dose LPS-induced epithelial damage by activating ERK1/2 to exert anti-apoptosis effects. Overexpression of TMEM16A had no effect on the proliferation of normal IEC-6 cells according to CCK-8 assay, but inhibited LPS-induced low cell proliferation. We predicted that TMEM16A might stimulate cell proliferation by activating ERK1/2 signaling pathways, and thus, protect against LPS-induced cell apoptosis. Low-dose and high-dose LPS-induced intestinal cell barrier dysfunction were caused by dysregulation of tight junctions and cell apoptosis, respectively. The negative effects of TMEM16A were not obvious during high-dose LPS-induced cell barrier dysfunction, suggesting that cell apoptosis might play a more important role in intestinal cell barrier dysfunction than tight junction dysregulation.

The current results provided some indication of the expression of TMEM16A and its effects on intestinal epithelial barrier function in normal and pathologically inflamed intestines. However, further studies are needed to clarify the different effects of tight junctions and cell apoptosis in the different stages of inflammatory intestinal diseases. TMEM16A may increase intestinal epithelial permeability in the initial stage of inflammation, but may then protect against inflammation-related cell apoptosis in the severe state of inflammation. However, it is difficult to determine the effects of TMEM16A on inflammation-related diseases such as IBD in vivo, because of the presence of other contributory factors in addition to cell loss and tight junctions, such as intestinal bacterial, epithelial cell secretion of MUCs, and antimicrobial peptides.

The expression but not the channel activity was involved to study the function of TMEM16A in intestinal cell barrier dysfunction in this study. Our previous results found that TMEM16A channel activity did not significantly affect LPS-induced barrier dysfunction (data not shown). Some studies also confirm that several TMEM16A inhibitors, which reduces TMEM16A currents does not inhibit cell proliferation^47^. These studies suggest that the protein expression of TMEM16A dominate the regulatory effect of cell proliferation compared with TMEM16A channel activity.

In conclusion, our findings demonstrated a significant increase in TMEM16A in an in vitro model of LPS-induced cell barrier dysfunction. The Ca^2+^-activated–Cl^−^ channel TMEM16A exerted a dual role in this model, and we speculated that its anti-apoptosis effects may be more important than its effect on tight junctions in terms of epithelial barrier dysfunction. However, further studies should be carried out to determine the effects of TMEM16A on intestinal epithelial barrier dysfunction in different intestinal inflammation-related diseases such as IBD in vivo.

## Supplementary information

Supplementary table and legends

Supplementary figure 1

Supplementary figure 1 legends
