# Supplementary material for: Dual role of Ca2+-activated Cl− channel transmembrane member 16A in lipopolysaccharide-induced intestinal epithelial barrier dysfunction in vitro
Source: Cell Death Dis. 2020 May 29;11(5):404. doi: 10.1038/s41419-020-2614-x (PMC7260209; doi:10.1038/s41419-020-2614-x)
Supplement: Supplementary file 1 — Supplementary table and legends [file 41419_2020_2614_MOESM1_ESM.docx]

Supplementary Table 1. Representative results from PROMO database for the prediction of transcription factors of TMEME16A

| Name | Number | Score |
| --- | --- | --- |
| NF-kappaB | T00590 | 10 |
| NF-kappaB | T00588 | 10 |
| GR | T00333 | 3 |
| FACB | T02841 | 3 |
| MF3 | T00507 | 5 |
| DSXF | T00955 | 7 |
| DSXM | T00956 | 7 |
| SPF1 | T03975 | 7 |
| GATA-1 | T00306 | 6 |
| NIT2 | T00627 | 7 |
| HOXA5 | T00377 | 5 |
| POU1F1a | T00691 | 5 |
| C/EBP delta | T00109 | 3 |
| unc-86 | T01882 | 5 |
| POU2F1 | T00643 | 6 |
| GATA-1 | T05705 | 6 |
| XBP-1 | T00902 | 6 |
| C/EBP beta | T00017 | 5 |
| GAMYB | T02679 | 8 |
| PRB | T00696 | 7 |
| PRA | T01661 | 7 |
| Dl | T00196 | 9 |
| HNF-3 beta | T01049 | 8 |
| YY1 | T00865 | 6 |
| YY1 | T00915 | 6 |
| POU2F1 | T01031 | 8 |
| MEF-2C/delta8 | T01769 | 11 |
| HNF-3 beta | T02344 | 7 |
| GR-alpha | T00337 | 5 |
| MYB2 | T02536 | 4 |
| B-factor | T00061 | 8 |
| HOXD8 | T01426 | 7 |
| HOXD8 | T01754 | 7 |
| Alfin1 | T04733 | 6 |
| HSF1 | T04394 | 5 |
| RC2 | T00724 | 6 |
| USF2 | T00878 | 10 |
| E47 | T05421 | 10 |
| Pax-6 | T00682 | 5 |
| NF-X3 | T01514 | 8 |
| T3R-beta | T00852 | 7 |
| VDR | T00885 | 7 |
| GA-BF | T00297 | 7 |
| NHP-1 | T00621 | 7 |
| WT1I-KTS | T00900 | 7 |
| WT1-KTS | T01839 | 7 |
| AP-2 alpha | T00033 | 9 |
| AP-2 alpha A | T00035 | 5 |
| MAZ | T00490 | 11 |
| AML1 | T01067 | 9 |
| MIG1 | T00509 | 11 |
| Fra-1 | T01208 | 6 |
| POU2F2(Oct-2.1) | T00646 | 8 |
| Eve | T00272 | 9 |
| C/EBP alpha | T00104 | 4 |
| Antp | T00026 | 13 |
| HNF-3 | T02277 | 6 |
| NF-AT4 | T01949 | 8 |
| HNF-3 beta | T02513 | 7 |
| SBF-1 | T00739 | 11 |
| GT-1 | T01089 | 11 |
| HNF-4 alpha1 | T00372 | 7 |
| HNF-1C | T01951 | 7 |
| Oct-B1 | T00545 | 8 |
| POU2F2B | T00662 | 8 |
| POU2F2B | T01871 | 8 |
| POU2F1a | T00644 | 9 |
| POU5F1(Oct-5) | T00653 | 9 |
| RXR-alpha | T01345 | 6 |
| POU5F1 | T00651 | 11 |
| POU2F1b | T01862 | 11 |
| POU2F1c | T01863 | 11 |
| GR-beta | T01920 | 5 |
| Nrf2:MafK | T05666 | 7 |
| STAT5A | T04683 | 12 |
| C/EBP alpha | T00105 | 6 |
| C/EBP beta | T00581 | 7 |
| C/EBP gamma | T00216 | 10 |
| IRF-2 | T00425 | 9 |
| DBP | T00183 | 7 |
| C/EBP alpha | T00108 | 3 |
| C/EBP beta | T00459 | 3 |
| Nkx2-1 | T00856 | 6 |
| GR | T00335 | 6 |
| AR | T00040 | 7 |
| Cutl1 | T02042 | 6 |
| HSF1(long) | T01042 | 8 |
| HSF1(short) | T02104 | 8 |
| Prd | T00699 | 7 |
| USF-1 | T00875 | 7 |
| WT1I | T01840 | 7 |
| IRF-1 | T00423 | 9 |
| GT-1 | T00339 | 8 |
| Nkx6-2 | T02050 | 8 |
| PF1 | T04784 | 8 |
| HNF-3alpha | T02512 | 10 |
| NF-AT1 | T00550 | 9 |
| FOXP3 | T04280 | 9 |
| AP-1 | T00029 | 9 |
| AP-1 | T00032 | 9 |
| POU1F1b | T01516 | 8 |
| POU1F1c | T01902 | 8 |
| c-Fos | T00124 | 7 |
| c-Fos | T00123 | 9 |
| MATa1 | T00488 | 7 |
| NF-1 | T00537 | 6 |
| NF-1 | T00539 | 6 |
| CREMtau | T01309 | 7 |
| CREMtau alpha | T01602 | 7 |
| CREMtau1 | T02108 | 7 |
| CREMtau2 | T02109 | 7 |
| YY1 | T00278 | 11 |
| ENKTF-1 | T00255 | 8 |
| Myogenin | T00528 | 7 |
| MyoD | T00526 | 8 |
| LEF-1 | T02905 | 8 |
| TCF-4E | T02878 | 7 |
| HNF-3alpha | T00371 | 8 |
| Nkx2-1 | T00857 | 7 |
| NF-E4 | T00560 | 10 |
| p53 | T00671 | 7 |
| CP2 | T00152 | 8 |
| NF-1 | T01298 | 8 |
| C1(long-form) | T01592 | 8 |
| c-Myb | T00137 | 8 |
| Pax-5 | T01201 | 9 |
| Tll | T00789 | 6 |
| CAT8 | T03227 | 12 |
| IPF1 | T02057 | 8 |
| COE1 | T01112 | 7 |
| ABF1 | T00056 | 6 |
| TCF-3 | T02857 | 11 |
| PEA3 | T00684 | 8 |
| STAT4 | T01577 | 6 |
| c-Ets-1 | T00112 | 7 |
| c-Ets-2 | T00113 | 6 |
| JunD | T00437 | 6 |
| c-Jun | T00131 | 6 |
| JunB | T00436 | 6 |
| STE12 | T00772 | 8 |
| c-Fos | T00122 | 7 |
| c-Jun | T00133 | 7 |
| Mad | T04378 | 5 |
| AREB6 | T00625 | 9 |
| Pax-1 | T00677 | 7 |
| Pax-2a | T00678 | 7 |
| Pax-2.1 | T03031 | 11 |
| NF-1(-like-proteins) | T00601 | 8 |
| Adf-1 | T00008 | 8 |
| NF-1/L | T00599 | 5 |
| LF-A1 | T00467 | 7 |
| HES-1 | T01649 | 7 |
| PIF3 | T04492 | 12 |
| MAT alpha2 | T00487 | 11 |
| USF-1 | T00877 | 11 |
| TFE3-S | T00814 | 12 |
| INSAF | T00406 | 8 |
| Pbx1b | T02088 | 11 |
| XPF-1 | T00906 | 9 |
| IA-1 | T05887 | 13 |
| Sp3 | T02419 | 11 |
| TCF-1(P) | T01109 | 10 |
| CDX2 | T03246 | 12 |
| TBP | T00794 | 8 |
| TFIID | T00820 | 8 |
| HSF1 | T00385 | 9 |
| Dfd | T00193 | 8 |
| HNF-1A | T00368 | 7 |
| HNF-1B | T01950 | 7 |
| DBP | T04875 | 7 |
| Crx | T03461 | 6 |
| Ftz | T00295 | 4 |
| p53 | T01806 | 8 |
| PBF | T02693 | 8 |
| mat1-Mc | T01275 | 9 |
| AbaA | T01085 | 10 |
| BTEB3 | T05051 | 9 |
| ADR1 | T00011 | 10 |
| POU4F1(l) | T01877 | 10 |
| HOXA4 | T00128 | 9 |
| En | T00253 | 11 |
| Elk-1 | T00250 | 8 |
| PU.1 | T02068 | 10 |
| Elk-1 | T05013 | 9 |
| c-Ets-1 | T00111 | 10 |
| GATA-1 | T00267 | 8 |
| GATA-1 | T00305 | 8 |
| USF2 | T02115 | 13 |
| PTF1-beta | T00701 | 10 |
| NF-Y | T00150 | 8 |
| NF-Y | T00613 | 8 |
| CTF | T00174 | 12 |
| CP1A | T00154 | 13 |
| C/EBP gamma | T02028 | 10 |
| c-Ets-2 | T01397 | 9 |
| MBP-1(1) | T00497 | 12 |
| SF-1 | T04014 | 8 |
| NFI/CTF | T00094 | 8 |
| RAR-beta:RXR-alpha | T05420 | 12 |
| Sp3 | T02338 | 9 |
| BTEB4 | T05053 | 9 |
| RAP1 | T00715 | 12 |
| Egr-3 | T00243 | 13 |
| Egr-3 | T05889 | 13 |
| C/EBP | T01388 | 7 |
| Smad3 | T04096 | 10 |
| Smad4 | T04292 | 11 |
| Zeste | T00918 | 7 |
| Zeste | T02100 | 7 |
| YY1 | T04970 | 9 |
| AR | T00042 | 10 |
| DTF-1 | T00201 | 11 |
| GCN4 | T00321 | 6 |
| CREM alpha | T01803 | 12 |
| CREB | T00164 | 10 |
| CRE-BP2 | T01017 | 8 |
| CREB beta | T02361 | 10 |
| AP-3 | T01150 | 9 |
| C/EBP alpha | T00107 | 8 |
| NF-1 | T00535 | 8 |
| LEF-1 | T00930 | 12 |
| GCM | T02302 | 9 |
| GCMa | T02306 | 9 |
| GCMa | T02307 | 9 |
| GCMb | T02308 | 9 |
| Nrf2 | T01443 | 11 |
| TAF-1 | T01090 | 12 |
| TRAB1 | T04821 | 12 |
| TRM1 | T05311 | 8 |
| SREBP-1c | T01562 | 10 |
| DPBF-1 | T04363 | 10 |
| DPBF-2 | T04364 | 10 |
| c-Myb | T00138 | 9 |
| AP-4 | T00036 | 11 |
| GATA-2 | T01302 | 8 |
| PEA3 | T00685 | 9 |
| MCB1 | T06035 | 9 |
| MCB2 | T06036 | 9 |
| HNF-6 | T03296 | 11 |
| AP-1 | T01140 | 7 |
| CPC1 | T00160 | 9 |
| GATA-3 | T00311 | 8 |
| Gt | T00328 | 11 |
| RAR-alpha1 | T00719 | 8 |
| RAR-beta | T00721 | 8 |
| T3R-alpha | T01351 | 10 |
| ER-beta | T04651 | 10 |
| T3R-beta1 | T00853 | 9 |
| IRF-2 | T01491 | 6 |
| CBF1 | T00080 | 8 |
| TOXE | T05645 | 10 |
| TAF | T00778 | 7 |
| NBF | T00951 | 9 |
| NBF | T01190 | 9 |
| AP-3(2) | T00039 | 10 |
| TCF-2 | T01110 | 10 |
| Opaque-2 | T00668 | 9 |
| CCBF | T00096 | 8 |
| SWI4 | T00775 | 8 |
| SWI6 | T01013 | 8 |
| SQUA | T03178 | 8 |
| DEF:GLO | T03216 | 9 |
| NF-AT2 | T01945 | 9 |
| NF-AT1 | T01948 | 8 |
| NF-AT1 | T01944 | 7 |
| RelA | T00594 | 10 |
| E2F | T01547 | 8 |
| STAT1beta | T01573 | 10 |
| c-Jun | T00132 | 9 |
| LBM1 | T05245 | 12 |
| NF-kappaB1 | T00593 | 11 |
| PUR-alpha | T05167 | 10 |
| PUR-beta | T05172 | 10 |
| LyF-1 | T00479 | 10 |
| HOXD9 | T01424 | 10 |
| HOXD10 | T01425 | 10 |
| HOXD9 | T01755 | 10 |
| HOXD9 | T01756 | 10 |
| HOXD10 | T01757 | 10 |
| HOXD10 | T01758 | 10 |
| PR-B | T00697 | 8 |
| GR | T05076 | 13 |
| LIM1 | T04817 | 10 |
| AP-2 | T00034 | 10 |
| MAC1 | T01265 | 7 |
| PU.1 | T00702 | 9 |
| Sp3 | T02453 | 13 |
| GAL4 | T00302 | 8 |
| HNF-3 | T00370 | 9 |
| COUP-TF1 | T00149 | 10 |
| ER-alpha | T00264 | 11 |
| ARP-1 | T00045 | 9 |
| ER-alpha | T00261 | 7 |
| AP-1 | T00031 | 11 |
| Crx | T03458 | 8 |
| RelA | T00595 | 11 |
| ELF-1 | T01113 | 13 |
| Yi | T00913 | 10 |
| RAR-gamma | T00720 | 8 |
| MNB1a | T01059 | 10 |
| Dof2 | T02690 | 10 |
| PPAR-alpha:RXR-alpha | T05221 | 11 |
| Ttk69K | T00843 | 8 |
| TCF-4 | T02918 | 10 |
| PXR-1:RXR-alpha | T05671 | 11 |
| FXR:RXR-alpha | T05318 | 12 |
| DEC2 | T05845 | 10 |
| SXR:RXR-alpha | T05670 | 9 |
| HNF-3beta | T03256 | 10 |
| PPAR-gamma:RXR-alpha | T05236 | 12 |
| Ik-1 | T02702 | 13 |
| NERF-1a | T05021 | 12 |
| SF-1 | T01147 | 9 |
| SF-1 | T02769 | 11 |
| T3R-alpha | T00841 | 12 |
| ATF-1 | T00968 | 12 |
| RXR-beta | T01332 | 12 |
| RXR-beta | T01349 | 9 |
| TBP | T00798 | 8 |
| MED8 | T03491 | 9 |
| HMGI(Y) | T02368 | 11 |
| GBF | T00315 | 11 |
| NF-AT4 | T01946 | 12 |
| NF-AT3 | T02462 | 10 |
| Fra-1 | T01462 | 13 |
| E74A | T00208 | 11 |
| GATA-2 | T00308 | 9 |
| SKO1 | T01306 | 9 |
| Myf-5 | T00521 | 13 |
| MEF-2DAB | T02505 | 11 |
| HNF-4alpha | T03828 | 13 |
| RFX1 | T01673 | 11 |
| INO2 | T01241 | 10 |
| POU2F1 | T00641 | 11 |
| POU2F2 | T00647 | 11 |
| POU2F2 | T00648 | 11 |
| POU2F2 (Oct-2.1) | T01864 | 11 |
| POU2F2 (Oct-2.3) | T01865 | 11 |
| POU2F2 (Oct-2.4) | T01866 | 11 |
| POU2F2 (Oct-2.6) | T01867 | 11 |
| POU2F2 (Oct-2.1) | T01870 | 11 |
| IPF1 | T02058 | 13 |
| Bcd | T00063 | 9 |
| f(alpha)-f(epsilon) | T00287 | 6 |
| ROX1 | T01286 | 10 |
| mec-3 | T01076 | 9 |
| Kr | T00456 | 11 |
| Pax-5 | T00070 | 7 |
| EBF | T05427 | 11 |
| HNF-4alpha | T05287 | 12 |
| HNF-4alpha2 | T02422 | 11 |
| HNF-4alpha1 | T02429 | 11 |
| Pbx1a | T01481 | 11 |
| HOXB1 | T01719 | 11 |
| COE2 | T05006 | 13 |
| COE3 | T05008 | 13 |
| E12 | T00204 | 13 |
| GCR1 | T00322 | 12 |
| NF-muNR | T01083 | 11 |
| Mitf | T01554 | 13 |
| SGF-3 | T00746 | 11 |
| ETF | T00270 | 13 |
| USF1 | T00874 | 8 |
| Tal-1 | T01799 | 11 |
| MTF-1 | T00515 | 13 |
| POU1F1 | T00707 | 10 |

Supplementary Table 2. Representative results from UCSC database for the prediction of transcription factors of TMEME16A.

| Matrix number | Name | Score | Relative score | Serial number | End | Forecast order |
| --- | --- | --- | --- | --- | --- | --- |
| MA0105.1 | NFKB1 | 10.8947 | 0.899 | 698 | 707 | GGGGATGTCC |
| MA0778.1 | NFKB2 | 4.39809 | 0.805 | 697 | 709 | TTGGACATCCCCC |
| MA0039.2 | KIf4 | 14.7301 | 0.987 | 804 | 813 | GGGGTGGGGC |
| MA1114.1 | PBX3 | 13.729 | 0.876 | 654 | 670 | TGATAAGTGACAGGGTG |
| PF0049.1 | CATTGTYY | 13.5321 | 0.991 | 272 | 279 | CATTGTCC |
| PH0012.1 | Cdx1 | 12.6394 | 0.882 | 1476 | 1491 | AGAGGTAATGAAACTT |
| PB0144.1 | Lef1_2 | 11.9858 | 0.873 | 674 | 689 | GGACATCAATAGACTT |
| MA0768.1 | LEF1 | 11.7191 | 0.845 | 959 | 973 | GAACATCAAACAAAT |
| PH0174.1 | Vax1 | 11.51 | 0.856 | 1477 | 1492 | AGTTTCATTACCTCTG |
| MA1114.1 | PBX3 | 11.4966 | 0.836 | 97 | 113 | CAGGGAGAGACAGGCTG |
| MA0848.1 | FOXO4 | 11.037 | 0.967 | 540 | 546 | ATAAACA |
| MA0848.1 | FOXO4 | 11.037 | 0.967 | 1293 | 1299 | ATAAACA |
| MA0848.1 | FOXO4 | 11.037 | 0.967 | 1393 | 1399 | ATAAACA |
| MA0039.2 | Klf4 | 11.0004 | 0.923 | 1985 | 1994 | TGGGTGCGGC |
| PB0040.1 | Lef1_1 | 10.6594 | 0.833 | 958 | 974 | GATTTGTTTGATGTTCA |
| PH0146.1 | Pou3f1 | 10.6412 | 0.803 | 1451 | 1467 | AATGAATGAATGAATGA |
| MA0463.1 | Bcl6 | 10.4731 | 0.849 | 1908 | 1921 | ATTACTAGAGAGAC |
| PB0144.1 | Lef1_2 | 10.3158 | 0.839 | 959 | 974 | GAACATCAAACAAATC |
| MA0039.1 | Klf4 | 10.1873 | 0.955 | 1539 | 1548 | AAGGGAAAGG |
| MA0039.2 | Klf4 | 10.0149 | 0.907 | 138 | 147 | AGGGTGAGGC |
| MA0039.2 | Klf4 | 9.9384 | 0.905 | 45 | 54 | GGGGTGGTGC |
| PB0034.1 | Irf4_1 | 9.62981 | 0.822 | 1474 | 1488 | GGTAATGAAACTTCT |
| MA0041.1 | Foxd3 | 9.5774 | 0.861 | 1391 | 1402 | ATTTGTTTATGC |
| MA1421.1 | TCF7L1 | 9.55427 | 0.814 | 959 | 970 | GAACATCAAACA |
| MA0846.1 | FOXC2 | 9.45869 | 0.881 | 1290 | 1301 | TGGATAAACACA |
| MA0039.1 | Klf4 | 9.43391 | 0.934 | 209 | 218 | AAGAGGAGGG |
| MA0039.2 | Klf 4 | 9.38264 | 0.896 | 295 | 304 | GAGGTGGGGC |
| MA1421.1 | TCF7L1 | 9.3822 | 0.811 | 467 | 478 | CACGGTCAAAGG |
| MA0846.1 | FOXC2 | 9.12533 | 0.876 | 1390 | 1401 | CGCATAAACAAA |
| PH0174.1 | Vax1 | 8.94571 | 0.805 | 1672 | 1687 | ATGGTCATTAGCCCTC |
| MA1421.1 | TCF7L1 | 8.85796 | 0.802 | 971 | 982 | AATCCTCAAAGA |
| PH0174.1 | Vax1 | 8.84463 | 0.803 | 1674 | 1689 | GGGCTAATGACCATGG |
| MA0041.1 | Foxd3 | 8.66053 | 0.842 | 1327 | 1338 | ATTCATTTGTTC |
| MA0041.1 | Foxd3 | 8.66053 | 0.842 | 1363 | 1374 | ATTCATTTGTTC |
| PB0115.1 | Ehf_2 | 8.63191 | 0.806 | 1176 | 1191 | TGAGAGTCCCTACCTT |
| MA0077.1 | SOX9 | 8.5105 | 0.871 | 677 | 685 | CTATTGATG |
| PB0011.1 | Ehf_1 | 8.47759 | 0.827 | 398 | 412 | AGGACCCCGAAGCAG |
| MA0039.2 | Klf4 | 8.46 | 0.880 | 1517 | 1526 | TGGGTGGGTA |
| PB0011.1 | Ehf_1 | 8.37168 | 0.825 | 1548 | 1562 | GTGCTGAGGAAGTGA |
| MA0041.1 | Foxd3 | 8.34403 | 0.836 | 1395 | 1406 | ATTGATTTGTTT |
| MA0077.1 | SOX9 | 8.28583 | 0.865 | 1400 | 1408 | GCATTGATT |
| PB0011.1 | Ehf_1 | 8.20281 | 0.821 | 1732 | 1746 | GTCAGCAGGAACTGT |
| MA0039.2 | Klf4 | 8.19733 | 0.876 | 631 | 640 | AGGTTGGGGC |
| PB0138.1 | Irf4_2 | 8.09496 | 0.820 | 227 | 241 | CTTTTTCTCAGGTGT |
| MA0463.1 | Bcl6 | 8.07914 | 0.811 | 1765 | 1778 | GTCCCTGGATGCCA |
| MA0041.1 | Foxd3 | 8.05354 | 0.830 | 64 | 75 | CTTTGTTTAGTT |
| MA0041.1 | Foxd3 | 8.0456 | 0.830 | 1339 | 1350 | GAATGTATGGTT |
| MA0077.1 | SOX9 | 8.02505 | 0.857 | 272 | 280 | GCATTGTCC |
| MA0039.1 | Klf4 | 8.0112 | 0.892 | 73 | 82 | AAGAGAAGAG |
| MA0039.1 | Klf4 | 7.95961 | 0.891 | 860 | 869 | GAAAGGAGGG |
| MA0039.1 | Klf4 | 7.86599 | 0.888 | 1847 | 1856 | TAGAGACAGG |
| MA0463.1 | Bcl6 | 7.76649 | 0.807 | 1138 | 1151 | CTCTCTGGAAGGTA |
| MA0041.1 | Foxd3 | 7.76569 | 0.824 | 962 | 973 | ATTTGTTTGATG |
| MA0039.1 | Klf4 | 7.75297 | 0.884 | 210 | 219 | AAAGAGGAGG |
| MA0039.1 | Klf4 | 7.71664 | 0.883 | 227 | 236 | TGAGAAAAAG |
| MA0039.1 | Klf4 | 7.66317 | 0.882 | 1540 | 1549 | GAAGGGAAAG |
| MA0846.1 | FOXC2 | 7.64017 | 0.853 | 64 | 74 | GAACTAAACAAA |
| MA0039.1 | Klf4 | 7.59953 | 0.880 | 1184 | 1193 | AGAAGGTAGG |
| MA0039.1 | Klf4 | 7.57233 | 0.879 | 1544 | 1553 | AAGTGAAGGG |
| MA0039.2 | Klf4 | 7.55323 | 0.865 | 429 | 438 | TGGCTGGGGT |
| MA0077.1 | SOX9 | 7.44658 | 0.841 | 1327 | 1335 | CATTTGTTC |
| MA0077.1 | SOX9 | 7.44658 | 0.841 | 1363 | 1371 | CATTTGTTC |
| PB0138.1 | Irf4_2 | 7.43231 | 0.802 | 1171 | 1185 | GGGACTCTCAGGGAG |
| MA0039.1 | Klf4 | 7.43226 | 0.875 | 859 | 868 | AAAGGAGGGG |
| PB0138.1 | Irf4_2 | 7.40463 | 0.802 | 1942 | 1956 | GCGGCTCTCAGACAG |
| MA0039.2 | Klf4 | 7.32892 | 0.861 | 417 | 426 | TGGGTGGGAA |
| MA0846.1 | FOXC2 | 7.28405 | 0.847 | 519 | 530 | TAAGGCAATATT |
| MA0039.1 | Klf4 | 7.2769 | 0.870 | 1202 | 1211 | TGGAAGAGGG |
| MA0039.1 | Klf4 | 7.17083 | 0.867 | 291 | 300 | TGGGGCAAGG |
| MA0039.2 | Klf4 | 7.0214 | 0.856 | 858 | 867 | AAGGAGGGGC |
| MA0039.1 | Klf4 | 6.98717 | 0.862 | 1422 | 1431 | TGAGGGAATG |
| MA0077.1 | SOX9 | 6.96398 | 0.827 | 1242 | 1250 | CTGTTGTTT |
| MA0077.1 | SOX9 | 6.93723 | 0.826 | 53 | 62 | CCATGGTCC |
| MA0041.1 | Foxd3 | 6.92323 | 0.808 | 223 | 234 | GTTTCTTTTTCT |
| MA0077.1 | SOX9 | 6.85228 | 0.824 | 496 | 504 | CCAGTGATC |
| MA0039.1 | Klf4 | 6.80906 | 0.857 | 437 | 446 | CAAAGAGGGG |
| MA0039.1 | Klf4 | 6.77099 | 0.856 | 861 | 870 | AGAAAGGAGG |
| PF0049.1 | CATTGTYY | 6.77039 | 0.871 | 68 | 75 | CTTTGTTT |
| PF0049.1 | CATTGTYY | 6.77039 | 0.871 | 537 | 544 | CAGTGTTT |
| PF0049.1 | CATTGTYY | 6.77039 | 0.871 | 1400 | 1407 | CATTGATT |
| MA0041.1 | Foxd3 | 6.76921 | 0.805 | 520 | 531 | AAATATTGCCTT |
| MA0039.1 | Klf4 | 6.76693 | 0.855 | 71 | 80 | CAAAGAGAAG |
| MA0039.1 | Klf4 | 6.76009 | 0.855 | 474 | 483 | AAAGGCAGAG |
| MA0039.2 | Klf4 | 6.73756 | 0.851 | 1756 | 1765 | AGGGTGAGGG |
| MA0077.1 | SOX9 | 6.73087 | 0.820 | 68 | 76 | TCTTTGTTT |
| MA0039.1 | Klf4 | 6.70372 | 0.854 | 1161 | 1170 | AGAGGAACGG |
| MA0039.1 | Klf4 | 6.69701 | 0.853 | 1538 | 1547 | AGGGAAAGGG |
| MA0041.1 | Foxd3 | 6.68707 | 0.803 | 524 | 535 | CAATATTTCATT |
| MA0039.1 | Klf4 | 6.67883 | 0.853 | 1354 | 1363 | TGAAAGTAGG |
| MA0039.2 | Klf4 | 6.66106 | 0.850 | 424 | 433 | GGGGTGGTGG |
| MA0041.1 | Foxd3 | 6.60193 | 0.801 | 966 | 977 | GAGGATTTGTTT |
| MA0039.1 | Klf4 | 6.56595 | 0.850 | 72 | 81 | AAAGAGAAGA |
| MA0039.2 | Klf4 | 6.50591 | 0.847 | 207 | 216 | GAGGAGGGGC |
| MA0848.1 | FOXO4 | 6.49405 | 0.877 | 678 | 684 | ATCAATA |
| MA0039.2 | Klf4 | 6.43669 | 0.846 | 1709 | 1718 | GGGGCGTGAG |
| MA0077.1 | SOX9 | 6.39138 | 0.810 | 926 | 934 | CTAGTGTTG |
| MA0039.1 | Klf4 | 6.33512 | 0.843 | 15 | 24 | TGAGGAGGGG |
| PF0049.1 | CATTGTYY | 6.29482 | 0.862 | 54 | 61 | CATGGTCC |
| MA0848.1 | FOXO4 | 6.21022 | 0.871 | 1968 | 1974 | GTAAACT |
| MA0039.1 | Klf4 | 6.19596 | 0.839 | 208 | 217 | AGAGGAGGGG |
| MA0077.1 | SOX9 | 6.16671 | 0.804 | 536 | 544 | TCAGTGTTT |
| MA0846.1 | FOXC2 | 6.09879 | 0.828 | 1241 | 1252 | TGAAACAACAGA |
| MA0039.1 | Klf4 | 6.08191 | 0.835 | 2091 | 2100 | ATGTGGAAGG |
| MA0039.1 | Klf4 | 6.05166 | 0.834 | 657 | 666 | AAGTGACAGG |
| MA0039.1 | Klf4 | 5.98386 | 0.833 | 454 | 463 | GAAAGGACGG |
| MA0039.1 | Klf4 | 5.93502 | 0.831 | 1188 | 1197 | GGGAAGAAGG |
| MA0039.1 | Klf4 | 5.9173 | 0.831 | 1624 | 1633 | AAGACTAAGG |
| MA0039.1 | Klf4 | 5.85252 | 0.829 | 624 | 633 | CAAAGCCAGG |
| MA0039.1 | Klf4 | 5.84457 | 0.828 | 67 | 76 | TAAACAAAGA |
| MA0039.2 | Klf4 | 5.84437 | 0.836 | 806 | 815 | TGGGGGTGGG |
| MA0039.1 | Klf4 | 5.77628 | 0.826 | 1418 | 1427 | TGAATGAGGG |
| MA0039.1 | Klf4 | 5.73416 | 0.825 | 1314 | 1323 | TGAATGAAAG |
| MA0039.1 | Klf4 | 5.68538 | 0.824 | 14 | 23 | CTGAGGAGGG |
| MA0846.1 | FOXC2 | 5.68054 | 0.822 | 928 | 939 | AACCTCAACACT |
| MA0039.1 | Klf4 | 5.64712 | 0.823 | 1167 | 1176 | AGGGAGAGAG |
| MA0039.1 | Klf4 | 5.61431 | 0.822 | 748 | 757 | TGGAGTAGGG |
| MA0039.1 | Klf4 | 5.60659 | 0.821 | 1549 | 1558 | TGAGGAAGTG |
| MA0039.1 | Klf4 | 5.59002 | 0.821 | 834 | 843 | CAGGGAGAAG |
| MA1125.1 | ZNF384 | 5.5145 | 0.826 | 224 | 235 | GAGAAAAAGAAA |
| MA0039.2 | Klf4 | 5.43043 | 0.829 | 1513 | 1522 | TGGGTAGGGG |
| MA0039.1 | Klf4 | 5.41639 | 0.816 | 1545 | 1554 | GAAGTGAAGG |
| MA0039.1 | Klf4 | 5.36761 | 0.814 | 806 | 815 | TGGGGGTGGG |
| MA0039.1 | Klf4 | 5.35236 | 0.814 | 1641 | 1650 | AAGAGCATGG |
| MA0848.1 | FOXO4 | 5.33769 | 0.854 | 66 | 72 | CTAAACA |
| MA0039.1 | Klf4 | 5.32993 | 0.813 | 100 | 109 | GAGAGACAGG |
| MA0039.1 | Klf4 | 5.23124 | 0.810 | 20 | 29 | AGGGGAGAAG |
| MA0039.1 | Klf4 | 5.2143 | 0.810 | 720 | 729 | CAGAGCAGAG |
| MA0039.1 | Klf4 | 5.17376 | 0.809 | 458 | 467 | GTGAGAAAGG |
| MA0039.1 | Klf4 | 5.13651 | 0.808 | 223 | 232 | AAAAAGAAAC |
| MA0848.1 | FOXO4 | 5.07087 | 0.849 | 1243 | 1249 | AACAACA |
| MA0039.1 | Klf4 | 5.06676 | 0.806 | 1326 | 1335 | TGAACAAATG |
| MA0846.1 | FOXC2 | 5.06595 | 0.812 | 1394 | 1405 | TAAACAAATCAA |
| MA0039.2 | Klf4 | 5.03554 | 0.822 | 691 | 700 | TGTGTGGGGG |
| MA0848.1 | FOXO4 | 5.01239 | 0.848 | 1740 | 1746 | GTCAGCA |
| MA0039.1 | Klf4 | 4.99794 | 0.804 | 1577 | 1586 | TGCAGGCAGG |
| MA0039.1 | Klf4 | 4.98283 | 0.803 | 1183 | 1192 | GAAGGTAGGG |
| MA0039.1 | Klf4 | 4.9492 | 0.802 | 1501 | 1510 | TCAGCAAAGG |
| MA0039.2 | Klf4 | 4.94258 | 0.820 | 689 | 698 | TGTGTGTGGG |
| MA0039.1 | Klf4 | 4.91475 | 0.801 | 140 | 149 | AGAGGGTGAG |
| MA0039.1 | Klf4 | 4.88247 | 0.800 | 769 | 778 | TAGAGGCTGG |
| MA0039.2 | Klf4 | 4.87889 | 0.819 | 891 | 900 | AGGGCTGAGC |
| MA0039.2 | Klf4 | 4.75031 | 0.817 | 616 | 625 | TGGGCAGAGC |
| MA0848.1 | FOXO4 | 4.7052 | 0.842 | 609 | 615 | GCCAACA |
| MA0848.1 | FOXO4 | 4.7052 | 0.842 | 1728 | 1734 | GCCAACA |
| MA1125.1 | ZNF384 | 4.70396 | 0.814 | 225 | 236 | TGAGAAAAAGAA |
| MA1125.1 | ZNF384 | 4.63536 | 0.813 | 65 | 76 | ACTAAACAAAGA |
| MA0846.1 | FOXC2 | 4.6264 | 0.805 | 538 | 549 | GCTATAAACACT |
| MA0039.2 | Klf4 | 4.61459 | 0.815 | 39 | 48 | TGGGCGGCAC |
| MA0846.1 | FOXC2 | 4.54463 | 0.804 | 1322 | 1333 | AGCATGAACAAA |
| MA0846.1 | FOXC2 | 4.47383 | 0.803 | 511 | 522 | TGACCAAATAAG |
| MA0846.1 | FOXC2 | 4.46041 | 0.803 | 1326 | 1337 | TGAACAAATGAA |
| MA0846.1 | FOXC2 | 4.34723 | 0.801 | 961 | 972 | ACATCAAACAAA |
| MA0848.1 | FOXO4 | 4.32533 | 0.834 | 1325 | 1331 | ATGAACA |
| MA0848.1 | FOXO4 | 4.32533 | 0.834 | 1464 | 1470 | ATGAACA |
| MA0039.2 | Klf4 | 4.29353 | 0.809 | 1871 | 1880 | AGAGTGGAGC |
| MA0846.1 | FOXC2 | 4.29219 | 0.800 | 1965 | 1976 | TTTGTAAACTCT |
| MA1125.1 | ZNF384 | 4.2289 | 0.807 | 223 | 234 | AGAAAAAGAAAC |
| MA0039.2 | Klf4 | 4.098 | 0.806 | 93 | 102 | AGGCTGGTGC |
| MA0039.2 | Klf4 | 3.98359 | 0.804 | 1632 | 1641 | GGGGCCTGGA |
| MA1125.1 | ZNF384 | 3.95725 | 0.802 | 962 | 973 | CATCAAACAAAT |
| MA0848.1 | FOXO4 | 3.95179 | 0.827 | 1838 | 1844 | GTCAAGA |
| MA0039.2 | Klf4 | 3.90746 | 0.803 | 1901 | 1810 | GGGGTCTGTC |
| MA0039.2 | Klf4 | 3.83045 | 0.802 | 356 | 365 | GGGGTGCTGT |
| MA0848.1 | FOXO4 | 3.71588 | 0.822 | 1125 | 1131 | AGCAACA |
| MA0848.1 | FOXO4 | 3.25984 | 0.813 | 930 | 936 | CTCAACA |
| MA0848.1 | FOXO4 | 3.20192 | 0.812 | 1319 | 1325 | GAAAGCA |
| MA0848.1 | FOXO4 | 2.92731 | 0.807 | 522 | 528 | GGCAATA |
| MA0848.1 | FOXO4 | 2.73281 | 0.803 | 817 | 823 | GTAAATG |
| MA0848.1 | FOXO4 | 2.73281 | 0.803 | 1086 | 1092 | GTAAATG |

Supplementary Table 3. Some representative results from JASPAR database for the prediction of transcription factors of TMEME16A.

| Name | Score |
| --- | --- |
| NFKB1 | 8 |
| Ahr::Arnt | 5 |
| Alx1 | 9 |
| Alx4 | 11 |
| Arid3a | 5 |
| Arid3b | 5 |
| Arid5a | 7 |
| Arnt | 6 |
| ARNT::HIF1A | 4 |
| Arntl | 6 |
| Ascl2 | 7 |
| Atf1 | 6 |
| Atf3 | 6 |
| Atoh1 | 3 |
| Bach1::Mafk | 11 |
| Barhl1 | 4 |
| Bcl6 | 8 |
| Bhlha15 | 3 |
| Bhlhe40 | 2 |
| CEBPA | 6 |
| CREB1 | 6 |
| Creb3l2 | 4 |
| Creb5 | 4 |
| Crx | 5 |
| Dlx1 | 4 |
| Dlx2 | 5 |
| Dlx3 | 5 |
| Dlx4 | 4 |
| Dmbx1 | 8 |
| Dux | 4 |
| E2F3 | 5 |
| EBF1 | 7 |
| Egr1 | 8 |
| EGR2 | 5 |
| ELF5 | 6 |
| En1 | 5 |
| Erg | 3 |
| Esrra | 10 |
| Esrrb | 5 |
| Esrrg | 4 |
| Ets1 | 6 |
| FOS::JUN | 6 |
| Foxa2 | 6 |
| Foxj2 | 4 |
| Foxj3 | 9 |
| Foxk1 | 7 |
| FOXO3 | 6 |
| Gabpa | 6 |
| Gata1 | 3 |
| Gata4 | 2 |
| Gfi1b | 4 |
| Hand1::Tcf3 | 6 |
| Hes1 | 5 |
| Hes2 | 6 |
| Hic1 | 4 |
| Hmx1 | 9 |
| HNF1B | 9 |
| Hnf4a | 12 |
| Hoxa11 | 5 |
| HOXA5 | 4 |
| Hoxa9 | 7 |
| Hoxc9 | 7 |
| Hoxd3 | 8 |
| Hoxd9 | 5 |
| Id2 | 4 |
| JUN::FOS | 6 |
| Klf1 | 6 |
| Klf12 | 7 |
| Klf4 | 4 |
| Lhx3 | 9 |
| Lhx4 | 3 |
| Lhx8 | 1 |
| Mafb | 6 |
| Meis1 | 7 |
| Mitf | 6 |
| Mlxip | 4 |
| Msx3 | 3 |
| Myb | 5 |
| Myc | 4 |
| Mycn | 4 |
| Myod1 | 2 |
| Myog | 2 |
| Neurog1 | 5 |
| NFATC2 | 7 |
| Nfe2l2 | 10 |
| NFYA | 9 |
| Nkx2-5 | 4 |
| Nkx2-5(var.2) | 3 |
| Nkx3-1 | 5 |
| Nkx3-2 | 6 |
| Nobox | 5 |
| Npas2 | 6 |
| Nr1h3::Rxra | 7 |
| Nr2e1 | 6 |
| Nr2e3 | 7 |
| NR3C1 | 8 |
| NR4A2 | 7 |
| Nr5a2 | 15 |
| Pax2 | 4 |
| Pdx1 | 1 |
| Phox2b | 7 |
| Pou2f3 | 11 |
| Pitx1 | 4 |
| Pou5f1::Sox2 | 11 |
| Pparg::Rxra | 11 |
| Prrx2 | 6 |
| Rarb | 9 |
| Rfx1 | 10 |
| Rhox11 | 11 |
| RUNX1 | 5 |
| Rxra | 3 |
| Shox2 | 3 |
| Six3 | 11 |
| Smad4 | 4 |
| SOX10 | 5 |
| Sox17 | 5 |
| Sox2 | 5 |
| Sox3 | 3 |
| Sox5 | 7 |
| Sox6 | 6 |
| SP1 | 7 |
| Spi1 | 8 |
| Srebf1(var.2) | 5 |
| Stat3 | 7 |
| Stat4 | 6 |
| Stat5a::Stat5b | 1 |
