# Supplementary figures and images for: Dual role of Ca2+-activated Cl− channel transmembrane member 16A in lipopolysaccharide-induced intestinal epithelial barrier dysfunction in vitro
Source: Cell Death Dis. 2020 May 29;11(5):404. doi: 10.1038/s41419-020-2614-x (PMC7260209; doi:10.1038/s41419-020-2614-x)

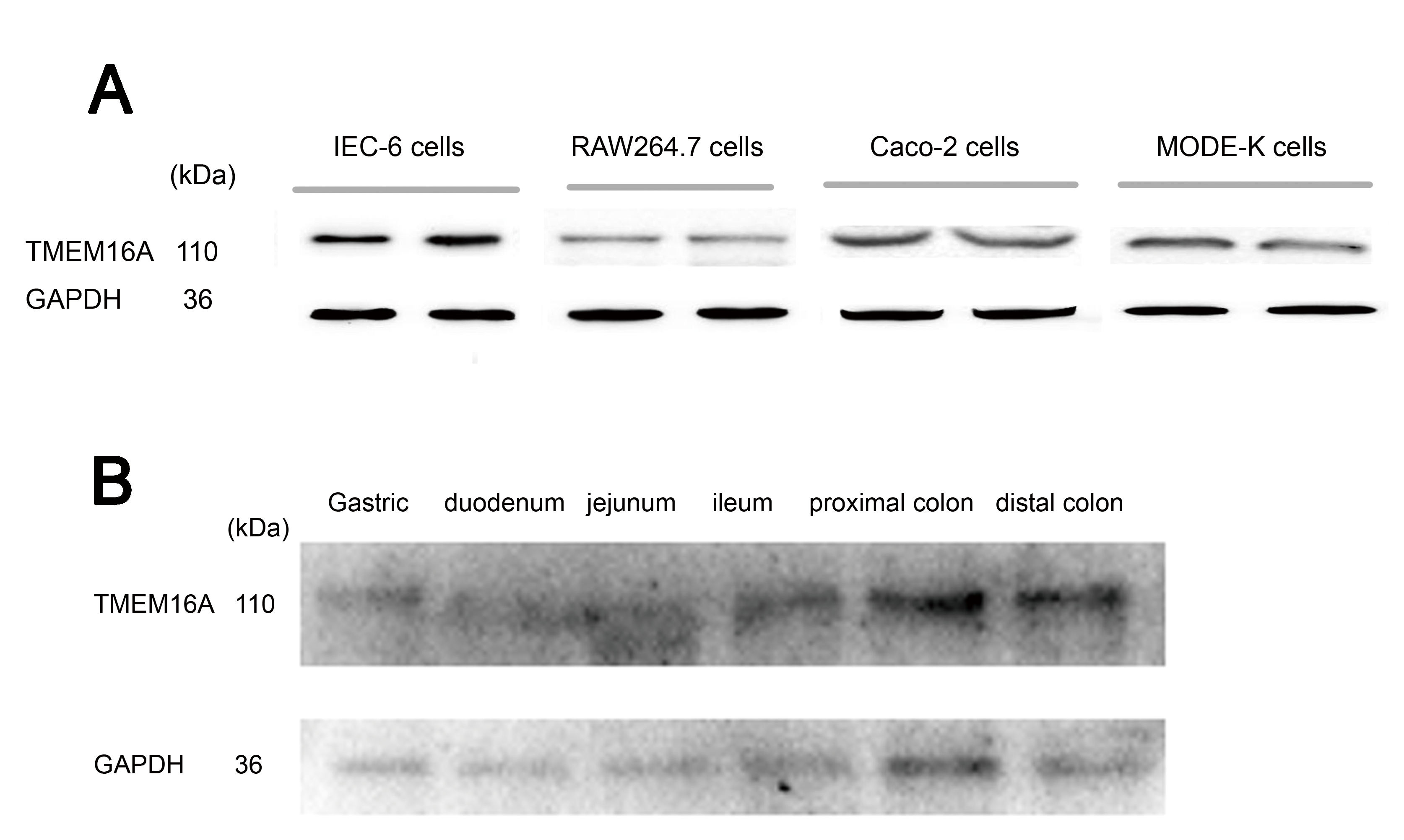

Supplement: Supplementary file 2 — Supplementary figure 1 [file 41419_2020_2614_MOESM2_ESM.tif]
