# Supplementary material for: Dual role of Ca2+-activated Cl− channel transmembrane member 16A in lipopolysaccharide-induced intestinal epithelial barrier dysfunction in vitro
Source: Cell Death Dis. 2020 May 29;11(5):404. doi: 10.1038/s41419-020-2614-x (PMC7260209; doi:10.1038/s41419-020-2614-x)
Supplement: Supplementary file 3 — Supplementary figure 1 legends [file 41419_2020_2614_MOESM3_ESM.docx]

As shown in supplementary figure 1A, the protein expression could be detected by Western blotting in IEC-6 cells, RAW264.7 cells, Caco-2 cells, and MODE-K cells respectively.

As shown in supplementary figure 1B, the protein expression profile of TMEM16A in gastric, small intestine and colon epithelium was observed.

**Supplementary figure 1**

Supplementary figure 1. A, the expression of TMEM16 in different kinds of cells; B, The expression of TMEM16A in gastrointestinal epitheliums.
